# Growth performance and survival of larval Atlantic herring, under the combined effects of elevated temperatures and CO_2_

**DOI:** 10.1371/journal.pone.0191947

**Published:** 2018-01-25

**Authors:** Michael Sswat, Martina H. Stiasny, Fredrik Jutfelt, Ulf Riebesell, Catriona Clemmesen

**Affiliations:** 1 GEOMAR Helmholtz Centre for Ocean Research Kiel, Kiel, Germany; 2 University of Kiel, Department of Economics, Kiel, Germany; 3 Norwegian University of Science and Technology, Department of Biology, Trondheim, Norway; 4 University of Gothenburg, Department of Biology, Kristineberg Centre for Marine Science, Fiskebäckskil, Sweden; Department of Agriculture and Water Resources, AUSTRALIA

## Abstract

In the coming decades, environmental change like warming and acidification will affect life in the ocean. While data on single stressor effects on fish are accumulating rapidly, we still know relatively little about interactive effects of multiple drivers. Of particular concern in this context are the early life stages of fish, for which direct effects of increased CO_2_ on growth and development have been observed. Whether these effects are further modified by elevated temperature was investigated here for the larvae of Atlantic herring (*Clupea harengus*), a commercially important fish species. Over a period of 32 days, larval survival, growth in size and weight, and instantaneous growth rate were assessed in a crossed experimental design of two temperatures (10°C and 12°C) with two CO_2_ levels (400 *μ*atm and 900 *μ*atm CO_2_) at food levels mimicking natural levels using natural prey. Elevated temperature alone led to increased swimming activity, as well as decreased survival and instantaneous growth rate (G_i_). The comparatively high sensitivity to elevated temperature in this study may have been influenced by low food levels offered to the larvae. Larval size, G_i_ and swimming activity were not affected by CO_2_, indicating tolerance of this species to projected "end of the century" CO_2_ levels. A synergistic effect of elevated temperature and CO_2_ was found for larval weight, where no effect of elevated CO_2_ concentrations was detected in the 12°C treatment, but a negative CO_2_ effect was found in the 10°C treatment. Contrasting CO_2_ effects were found for survival between the two temperatures. Under ambient CO_2_ conditions survival was increased at 12°C compared to 10°C. In general, CO_2_ effects were minor and considered negligible compared to the effect of temperature under these mimicked natural food conditions. These findings emphasize the need to include biotic factors such as energy supply via prey availability in future studies on interactive effects of multiple stressors.

## Introduction

Ocean warming (OW) and acidification (OA), both caused by rising atmospheric CO_2_ levels [1,2], will intensify in the future. Sea surface temperature is projected to increase by at least 1.5°C while seawater pH could decrease by 0.4, when pCO2 levels of ~800 μatm are reached by the year 2100 [3,4]. These environmental changes will likely interact in their effects on fish populations and are therefore of great interest to society due to the socio-economic value of fishing and other ecosystem services [5,6]. The early developmental stages of fish (eggs and larvae) are of particular importance for ecosystems and fisheries since they represent a critical bottleneck for recruitment [7,8]. The performance of fish species and their developmental stages should be best around their abiotic and biotic optimum [9]. The theoretical optimal temperature of a species corresponds to optimal [10] or even suboptimal [11] growth temperatures and may interact with future CO_2_ concentrations, as observed for larval European sea bass (*Dicentrarchus labrax*) [12] and Senegalese sole (*Solea senegalensis*) [13]. Larval European sea bass displayed higher survival at increased temperatures and elevated *p*CO_2_ levels, leading to heavier juveniles in the warm treatment and lower aerobic scope under elevated *p*CO_2_ levels [12]. In contrast, the early life stages of Senegalese sole showed strong negative impacts of increased *p*CO_2_ and temperatures on hatching success and survival, while larval growth and metabolism were positively affected by increased temperature but negatively affected by increased *p*CO_2_ [13]. A synergistic effect of elevated temperature and *p*CO_2_ was detected as an increase in skeletal deformities and otolith size [13]. Although few studies have tested the interaction of increased temperature and *p*CO_2_ for the early life stages of fish, these two exemplary studies highlight the complexity in the interacting effects of temperature and *p*CO_2_.

Considerably more information is available on the single effect of increased *p*CO_2_ on early developmental stages of fish, which, in general, are thought to be most susceptible to changes in pH and therefore most vulnerable to OA [14,15]. This can be attributed to the fact that gills, the main organ for pH regulation, are not yet functional during early larval development and thus larvae have to rely on other regulatory mechanisms (e.g. via cutaneous chloride cells) [16,17]. Detrimental effects of elevated CO_2_ levels (e.g. organ damage [18] and increased embryonic mortality [19,20]) have been reported for the larvae of a range of fish species. Impacts of OA on behaviour [21,22] were related to an interaction between CO_2_ and neurotransmitter function [23]. However, few or no effects of OA have been found for the larvae of several other species for several parameters [24–28], demonstrating that larval responses may be species specific.

Intraspecific differences in the effect of OA have also been reported, for example, in the early developmental stages of Atlantic herring (*Clupea harengus*) one of the most important economic fish species in the North Atlantic [29]. No evidence of negative OA impacts, even at 4600 μatm CO_2_, was found for Baltic Sea herring eggs and larvae at hatch on several parameters like hatch rate and size. However, nutritional condition in terms of RNA:DNA [30] and egg survival [31] was found to be negatively affected. In older larvae of Norwegian spring-spawning herring, in contrast, a negative effect on growth, development, condition and tissue formation was observed at CO_2_ levels >1800 μatm CO_2_ [32], while swimming kinematics and proteome structure were unaffected [33,34]. As a coastal bottom-spawning species the eggs and newly hatched larvae of herring can already encounter CO_2_ levels higher than expected for the end of the century, due to the seasonal variation and biological activity in coastal areas, especially in the Baltic Sea [35,36], which could explain the above mentioned differences in response to OA in performance between populations. It is therefore important to study these variable responses in relation to temperature and *p*CO_2_ changes to better understand the potential effects on recruitment of fish populations in a future ocean.

In this study, survival and performance of larval Atlantic herring was investigated under ambient and elevated CO_2_-concentrations (400 and 900μatm *p*CO_2_) within two temperature regimes (10 and 12°C). We developed three hypotheses to test in this study: 1. Elevated CO_2_ levels negatively affect length, weight, instantaneous growth rate, development and survival, while increasing swimming activity; 2. These traits are negatively affected in the higher temperature treatment due to the natural food conditions; and 3. This leads to a synergistic negative effect of elevated CO_2_ concentrations and higher temperatures.

## Materials and methods

### Experimental set-up

The experiment was performed at the Sven Lovén Centre for Marine Sciences in Kristineberg, Sweden from April to June 2013. The brood stock herring originated from the Oslo-Fjord close to the Biological Station Drøbak, University of Oslo and were caught using a gillnet on April, 22^nd^ 2013 at a depth of ~30m, at the southern tip of Søndre Kaholmen, roughly located at 59°40'29" N and 10°36'22" E. The dead, ready-to-spawn herring were transported on ice to the Sven Lovén Centre, where fertilization was performed four hours later. The eggs from five females were stripped separately on 150 plastic plates with an area of 6 cm^2^ and ~150 eggs per plate. Subsequently, the sperm from five males was used to create a total of 25 distinct families, each on six egg plates. Fertilization was performed in seawater of the two CO_2_ treatments (~400 μatm and ~900 μatm CO_2_), already in line with the experimental CO_2_ treatment of the larvae after hatch to include possible effects on sperm motility and fertilization success. To synchronize the time of hatching, egg incubation was performed at ambient CO_2_ (400 μatm) conditions and 10°C. Herring have an egg development of ~130 degree days, which means 13 days at 10°C and 11 days at 12°C. This two day difference in hatching would have meant variation in prey levels per day. Different times of hatching would thus have been problematic to directly relate differences between larvae to treatment effects.

### Larval rearing

**Fig 1:**
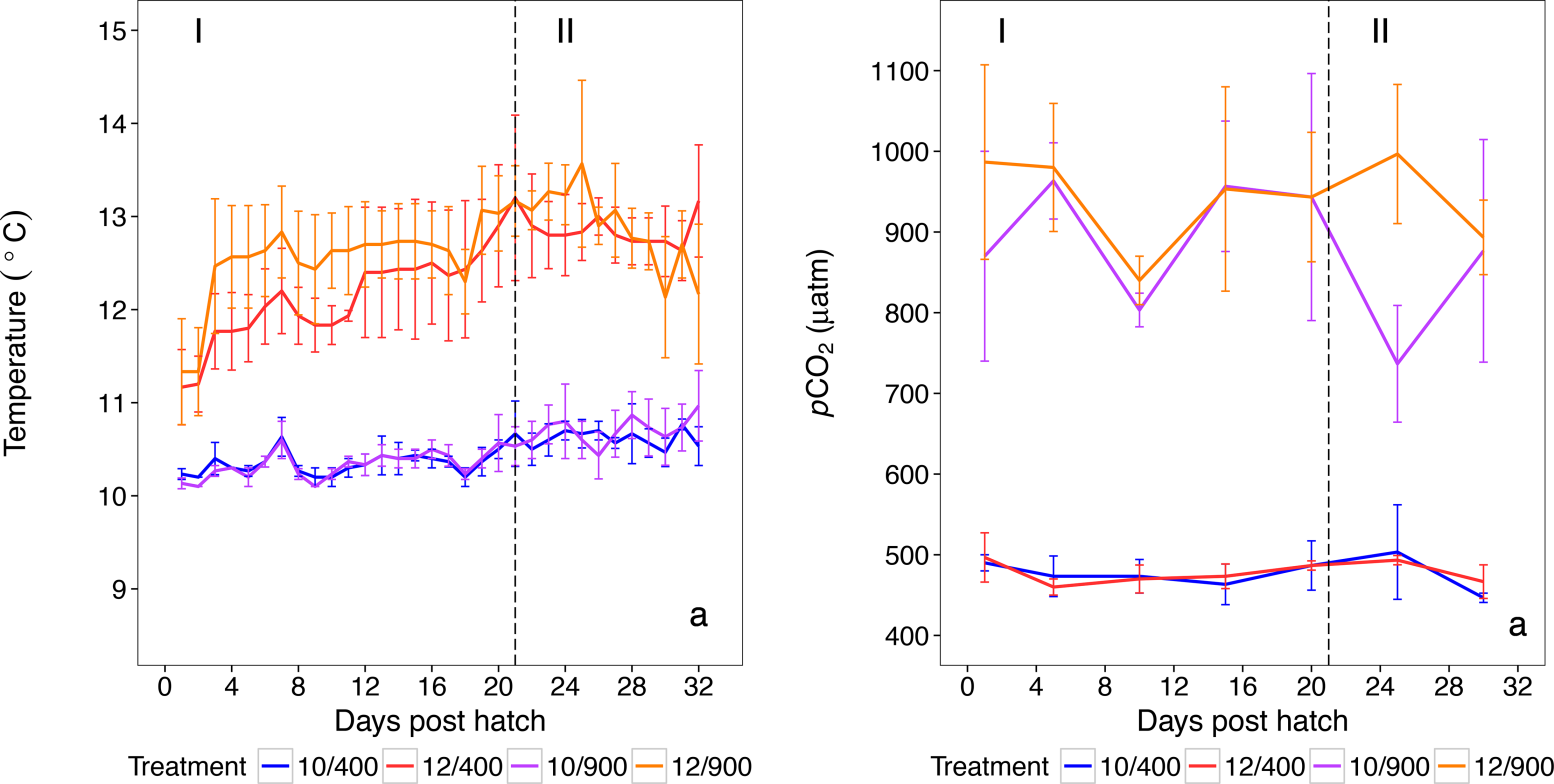
**Temperature (a) and measured *p*CO_2_ (b) per treatment combination (mean and standard error) over the experimental period against days post hatch**.

For the larval rearing two temperatures (10 and 12°C) and two CO_2_-levels (ambient: 400 μatm and elevated: 900 μatm CO_2_) were fully crossed with 3 replicates, resulting in twelve tanks in total. The treatments are hereafter named according to their temperature and CO_2_ conditions: 10/400, 10/900, 12/400 and 12/900. The two temperature treatments are within the preferred range (8–12°C) of Atlantic herring larvae described by Mehner et al. [37]. At the time of peak hatch (more than 50% of larvae hatched) the larvae were distributed randomly into plastic bags, which were lowered into the rearing tanks to allow for temperature acclimatization prior to introduction. Larvae were kept at the CO_2_ level at which the eggs had been fertilized, but were randomly distributed between the replicates. The rearing water was taken from the fjord (inlet at 30 m depth); temperature was adjusted before UV treatment and filtration (pore size 500 μm, 50 μm, 20 μm and 5 μm). The treated water was distributed to the rearing tanks of 90 L via twelve individual header tanks of 50 L, where CO_2_ manipulation took place. The pH probes were installed in the header tanks, which were connected to a computer-based pH-manipulation-system (IKS COMPUTERSYSTEME GMBH). The IKS system constantly measured pH in the header tanks. If the system detected deviations from the set target pH of 7.8 for the high CO_2_ treatments, CO_2_ was injected into the header tanks via opening of a magnetic valve. Additional air bubbling in the header tanks assured mixing and air-water oxygen equilibration. Constant bubbling with tiny pores provided gentle stirring of the rearing tanks, and the daily siphoning of dead prey items and other particles ensured a clean environment for the larvae. Mean water flow-through rate was 200 ml/min per individual header and respective rearing tank, leading to a renewal time of water per rearing tank of ten hours. The light was set according to natural, ambient hours of daylight (16 to 17 hours per day with mean light intensities of 18 μE). Temperature and pH were measured in each tank daily (WTW pH/Cond 340i/3320, WTW Wissenschaftlich-Technische Werkstätten GmbH), while *p*CO_2_ was measured every five days using a method described by Green and Jutfelt [38] using infra-red CO_2_ absorbance (Vaisala, Helsinki, Finland). Temperature, pH and CO_2_ measurements were taken at the same time by lowering the probes in the rearing tank approximately 5cm below the water surface. The probes were left inside the tank until values were stable. Measurements were taken in the morning before feeding and syphoning, but pH was checked occasionally during the day to assure stable conditions. Temperature and measured *p*CO_2_ over time are shown in (Fig 1A and 1B). *p*CO_2_ was additionally calculated at the beginning and the end of the experiment using the free software CO2SYS [39] with the set constants of [40] refitted by Dickson & Millero [41] and the measured parameters dissolved inorganic carbon (DIC) and total alkalinity (Table 1) based on the Best Practices Guide [42].

**Table 1:** Abiotic factors per treatment combination (T = temperature, pH = -log_10_[hydrogen ions], TA = total alkalinity, DIC = dissolved inorganic carbon, CO_2_ m = measured CO_2_, CO_2_ c = calculated CO_2_ (means and standard deviations derived from the various measurements over the experimental time).

Treatment	T (°C)	pH (units)	TA (μmol/kg)	DIC (μmol/kg)	CO_2_ m (μatm)	CO_2_ c (μatm)
10/400	10.4 ± 0.2	8.11 ± 0.05	2320 ± 19	2156 ± 6	475 ± 20	450 ± 58
10/900	10.5 ± 0.3	7.81 ± 0.10	2304 ± 4	2257 ± 12	877 ± 68	982 ± 23
12/400	12.4 ± 0.7	8.10 ± 0.06	2328 ± 11	2128 ± 51	479 ± 15	463 ± 59
12/900	12.7 ± 0.6	7.79 ± 0.08	2328 ± 10	2259 ± 6	938 ± 44	907 ± 61

**Fig 2:**
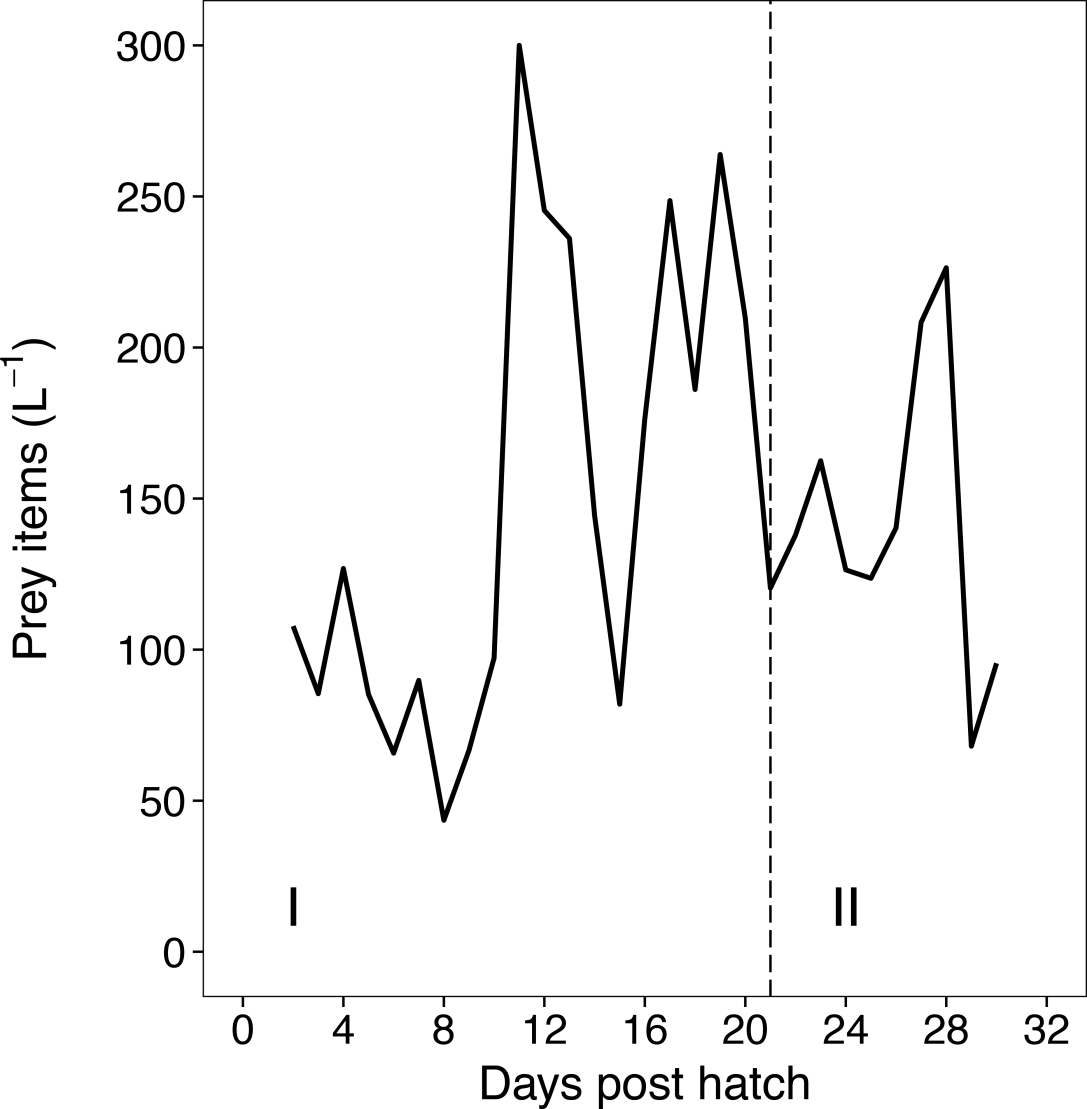
Calculated daily prey densities (in the size range from 70–200μm consisting mainly of calanoid copepods) offered to herring larvae over the experimental period in days post hatch.

**Fig 3:**
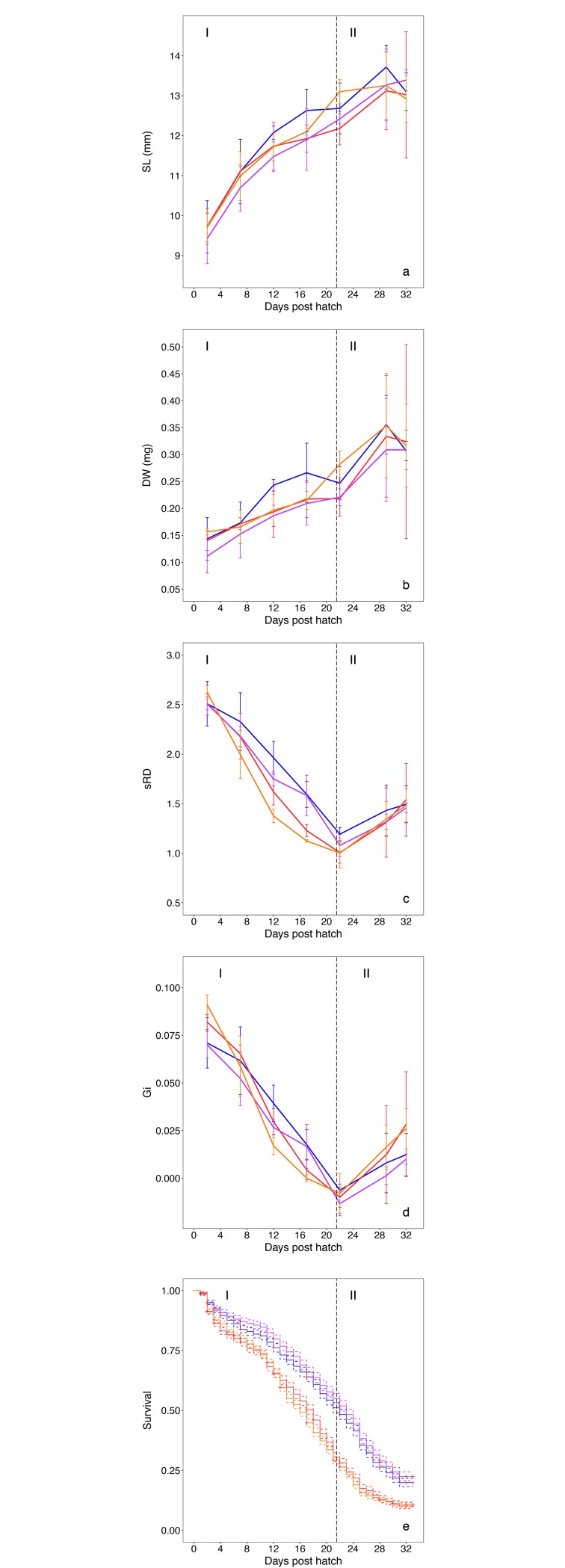
**Herring larvae performance per treatment combination of two temperatures, 10°C and 12°C, and CO_2_ levels, 400μatm and 900μatm *p*CO_2_, (mean ± standard deviation) for the whole experimental period against days post hatch**: a) standard length SL (mm), b) dry weight DW (mg), c) nutritional condition sRD, d) instantaneous growth rate G_i_ and e) survival. The vertical line represents the separation into phases I and II.

Herring larvae were fed three times per day with natural plankton pumped from the fjord and concentrated with a Hydrotech plankton filter. The prey consisted mainly of calanoid copepod nauplii and copepodites with a size range of 70–200 μm, which are natural prey for herring larvae [43–46]. Per day, mean prey densities of 147 items L^-1^ (minimum 44 and maximum 300 prey items L^-1^ at 8 DPH (days post hatch) and 11 DPH, respectively) of rearing water were achieved in each tank (Fig 2) and grazed down till the next feeding event. Mean prey densities were above the critical prey density for herring, estimated as 10 prey items L^-1^ [47,48]. Laurence [49] describes a natural range of 5–80 nauplii L^-1^, which defines this study to be close to natural prey densities and considerably lower than in other studies with ~2000 prey items L^-1^ [32,50]. The low prey densities are also depicted by low standardized RNA/DNA ratios (sRD) during the study, a proxy for larval nutritional condition, (Fig 3C). Additionally a solution of a green algae *Nannochloropsis sp*. (6*10^5^ cells L^-1^) was given to create "green water" conditions during the whole experiment, which were shown to have a positive effect on fish larvae [51], also recently shown for first feeding herring [52].

### Sampling & analysis

Each morning the dead larvae were collected by siphoning the tank floors and counted to calculate mortality rates. Sampling was conducted approximately every five days. After anaesthetization with tricaine methanesulfonate (MS-222) and before storage at minus 80°C, a photograph of each individual larva was taken with a digital camera attached to a stereomicroscope (Olympus SZX 7 with Olympus DP 26 Camera and Olympus Stream Essentials Software). Standard length (SL) measurements were taken from calibrated photos of each larva with the open source software Image J [53]. Developmental stages were determined according to Doyle [54] and the proportional contribution of each developmental stage was calculated. Dry weight (DW) was measured for each individual larva on a micro balance (Sartorius SC 2 micro balance, Sartorius AG, Göttingen, Germany; precision ±0.1 μg) after freeze drying (Christ Alpha 1–4 freeze dryer, Martin Christ Gefriertrocknungsanlagen GmbH, Osterrode, Germany). The same larvae were later used for nucleic acid analysis according to the protocol described in Malzahn et al. [55]. As an indicator of nutritional condition, the ratio of the nucleic acids RNA and DNA (RD), defined as the number of protein biosynthesis machinery per cell was standardized to sRD according to Caldarone et al. [56]: *sRD* = 0.92 × *RD*. Since different temperatures can lead to different activities of these protein biosynthesis units, different estimated growth rates resulting from the total amount of RNA were expected. To compare resulting growth rates from the two temperature treatments, dry weight related instantaneous growth rates (G_i_) of the larvae were calculated [57] based on the following relationship:

$$G_{i}=0.0145*sRD+0.0044*(sRD*T)-0.078$$

With G_i_ being the instantaneous growth rate, sRD the intercalibrated standardized RNA/DNA ratio and T the temperature determined at the sampling time. A resulting G_i_-value of 0 would mean no growth at all and a value of 1 resembles the doubling of the weight of the larva per day. Details on the number of larvae analysed per parameter are given in S1 Table. The swimming activity was measured for the survivors by automated video tracking software similar to the methods described in detail in Sundin & Jutfelt [58]. Briefly, six larvae per tank were carefully transferred from the rearing tanks into individual circular chambers (diameter: 35 mm, 10 mm water height). As the larvae are transparent the chambers were placed on a black background and lit by light ramps from the sides, making the larvae appear white. This provided sufficient contrast for reliable tracking of larval movements. The larvae were left undisturbed for 20 min, during which time they were monitored using a firewire camera (Dragonfly 2, Pointgray, Richmond BC, Canada), connected to a computer mounted above the chambers. The software (Zebralab, Viewpoint, Lyon, France) measured all movements of each larva (all movements over a certain threshold to avoid image noise being counted as larval movements). The sampling frequency was 30 frames per second. The software calculated the integrated swimming activity per minute, meaning all motion was detected during this time, which was then averaged for each larva during the 20 minutes interval and used for further analysis (S1 Fig).

The experiment was divided into two phases based on the nutritional condition (sRD) of the larvae at a time before and after 22 DPH, where a point of inflexion in sRD was observed (Fig 3). Phase I is characterized by a decline, while phase II shows an increasing trend in sRD. To relate larval age in DPH to the larval development we chose to state degree days for selected events, which is the cumulative sum of mean daily temperatures for a given age in DPH, according to Fuiman et al. [59] the best comparison of ontogenetic stages within a species.

In the 12/400-treatment a high mortality event in one of the replicate tanks occurred at 21 DPH. The remaining larvae of this tank were sampled until the end of the experiment. In contrast to the two other replicates from 12/400, this tank experienced a strong increase in length, weight and nutritional condition in the second phase, which indicates an effect of this high mortality event, probably due to increased food availability per each individual larva. Since the causes for the higher mortality are unknown and triplicated treatments do not allow for an identification of outliers, the data from this tank are therefore included in the analyses.

For statistical analyses of growth and development over time, a repeated-measures ANOVA was applied as a linear-mixed-effect model. Here, the two experimental treatments "Temperature" and "CO_2_" as well as "Time" in DPH were used as fixed factors. By choosing "Tank number" as a random factor, dependency of measurements at different time points was taken into account as well as possible "tank-effects". The models were fitted to the data using means of standard length, dry weight and G_i_ and developmental status per tank and sampling day. Mean developmental status was calculated by attributing continuous numbering to the "Doyle stages", i.e. the Doyle stage 1a = 1, 1b = 2, …, 3b = 8. For data analysed by the repeated-measures-ANOVA approach (linear-mixed-effect models) F- and p-values as well as degrees of freedom (DF) are given (Table 2, S2 Table). Table 2 only shows the significant factors, while in the supplement S2 Table all factors are listed.

**Table 2:** Results from repeated-measures ANOVA models. Only significant factors are shown for the different parameters with the respective F-values and p-values. The factors listed are additive for the described models. The complete table can be found in S2 Table.

Parameter	Period	Temperature	Factor	F-value	p-value
Length	Whole	10°C / 12°C	Time	244.78	<0.0001
	Phase 1	10°C / 12°C	Time	192.61	<0.0001
	Phase 2	10°C / 12°C	Time	6.61	<0.05
Weight	Whole	10°C / 12°C	Time	199.63	<0.0001
	Phase 1	10°C / 12°C	Time	89.20	<0.0001
			Temp*CO_2_	10.99	<0.05
	Phase 2	10°C / 12°C	Time	13.66	<0.05
Instantaneous growth rate	Whole	10°C / 12°C	Time	89.25	<0.0001
	Phase 1	10°C / 12°C	Time	609.72	<0.0001
			Temp*Time	7.41	<0.01
	Phase 2	10°C / 12°C	Time	60.78	<0.001
Swimming activity	Final	10°C / 12°C	SL	25.90	<0.001
			Temp	7.12	<0.01
			SL*Temp	5.42	<0.05
Development	Whole	10°C / 12°C	Time	678.71	<0.0001
	Phase 1	10°C / 12°C	Time	617.78	<0.0001
			Temp*Time	6.560	<0.05
	Phase 2	10°C / 12°C	Time	14.50	<0.01

Swimming activity was measured at the end of the experiment. For the statistical analysis of the activity patterns, the averaged swimming activity of each single larva was used in an ANCOVA approach to include the additional covariate standard length. The assumptions of an ANCOVA, homogeneity of variance, normality and independency of covariates, were assessed visually. The respective likeliness of fit (R^2^), DF, F and p-values are displayed (Table 2, S3 Table). In both approaches, repeated-measures ANOVA and ANCOVA, the best model was detected by searching for the smallest Akaike Information Criterion, if differences compared to the more complex models were not significant. For the survival analysis Cox proportional hazards model was chosen (Table 3), with exp(coef) being the ratio between mean survival of one treatment group to another and therefore showing which group has a better survival. Exp(coef)>1 in this case means that the described group (treatment combination) shows a lower survival than the reference group. The survival analysis was repeated for both temperatures separately to discriminate between combined effects of temperature and CO_2_. The significance level for all statistical analysis was set to p<0.05. The statistical analyses were performed for the whole experimental period and for phase I and phase II separately, based on the above mentioned distinction in nutritional condition (sRD).

**Table 3:** Results of the Cox-PH models for survival with the respective Chi squares, degrees of freedom (DF), p-values and approximate hazards (exp(coef)). Treatment values of 12/900 refer to °C/μatm CO_2_ and the given interaction.

Parameter	Temperature	Period	Chi	DF	Treatment	p-value	exp(coef)
Survival	10°C & 12°C	Whole	488.6	3	**12**	**<0.001**	**1.54**
					**900**	**<0.05**	**0.92**
					12*900	**<0.01**	**1.13**
		Phase I	36.29	3	**12**	**<0.05**	**1.09**
					900	0.18	0.94
					12*900	**<0.05**	**1.13**
		Phase II	17.84	1	**12**	**<0.001**	**1.21**
	10°C	Whole	6.2	1	**900**	**<0.05**	**0.92**
		Phase I	1.81	1	900	0.18	0.94
		Phase II	0.69	1	900	0.41	0.95
	12°C	Whole	1.8	1	900	0.20	1.04
		Phase I	3.95	1	**900**	**<0.05**	**1.06**
		Phase II	0.69	1	900	0.41	0.94

### Ethics statement

Animal welfare was assured by performing the experiment according to the guidelines of the ethics permit (number 332–2012) with a separate permit for the behavioural part of the study (number 151–2011), both issued by the Swedish Board of Agriculture "Jordbruksverket"). In order to minimize stress, specimens were anaesthetized using MS-222 before handling and fixation. The species (*Clupea harengus*) used is not endangered and was obtained from a local registered and licensed fisherman (license ID = 977 224 357).

## Results

### Effect on larval size

Herring larvae started with a mean SL of 9.6 mm ± 0.5 SD at 2 DPH and reached 13.1 mm ± 0.8 SD after 32 DPH (Fig 3A). The increasing size with age was validated by the significant effect of time (Table 2). Neither temperature nor CO_2_ alone, or in combination, showed a statistically significant effect on larval size (Table 2)(Fig 3A).

### Effect on larval dry weight

Mean larval DW increased over time from 0.14 ± 0.03 mg at 2 DPH to 0.31 ± 0.09 mg at 32 DPH (Fig 3B). A significant interaction effect of temperature and CO_2_ on DW was detected for phase I (p<0.05). This interaction of temperature and CO_2_ on DW, however, was not significant for phase II or the whole period (Table 2). The combined effect of temperature and CO_2_ in phase I was visible at 10°C with higher DW in the ambient compared to the high CO_2_ treatment, which could not be seen at 12°C.

### Instantaneous growth rate

The development of G_i_ over time is represented by values of 0.093 ± 0.028 at 2 DPH, -0.003 ± 0.022 at 22 DPH and 0.028 ± 0.027 at 32 DPH (Fig 3D). In phase I when G_i_ was declining, temperature was found to have a significant effect over time with in general lower G_i_ values at 12°C (Table 2). During this phase no significant CO_2_ effect was detected (S2 Table). From 22 DPH (10°C = ~225 degree days; 12°C = ~270 degree days) when G_i_ values were lowest until the end of the study, G_i_ values were increasing (phase II) with a stronger increase at 12°C (p = 0.05) (S2 Table).

### Survival

The larval survival displayed a steady decline until the end of the experiment (Fig 3E). Survival was significantly lower in 12/400 and 12/900 than in 10/400 and 10/900 for the whole period and in each separate phase (Table 3). A significant interaction of temperature and CO_2_ was found for the whole period and phase I. Higher survival was detected in 10/900 than in 10/400 over the whole period, though there were no significant effects in the single phases I and II. 12/400 showed significantly higher survival than 12/900 only in phase I, but not for the whole period and phase II (Table 3). The difference in survival between CO_2_ treatments was in the range of 1–2 days, while ~6 days separate the two temperature treatments. Also confidence intervals depicted the difference between CO_2_ treatments in 10°C as small compared to the effect of temperature (Fig 3E).

### Swimming activity

**Fig 4:**
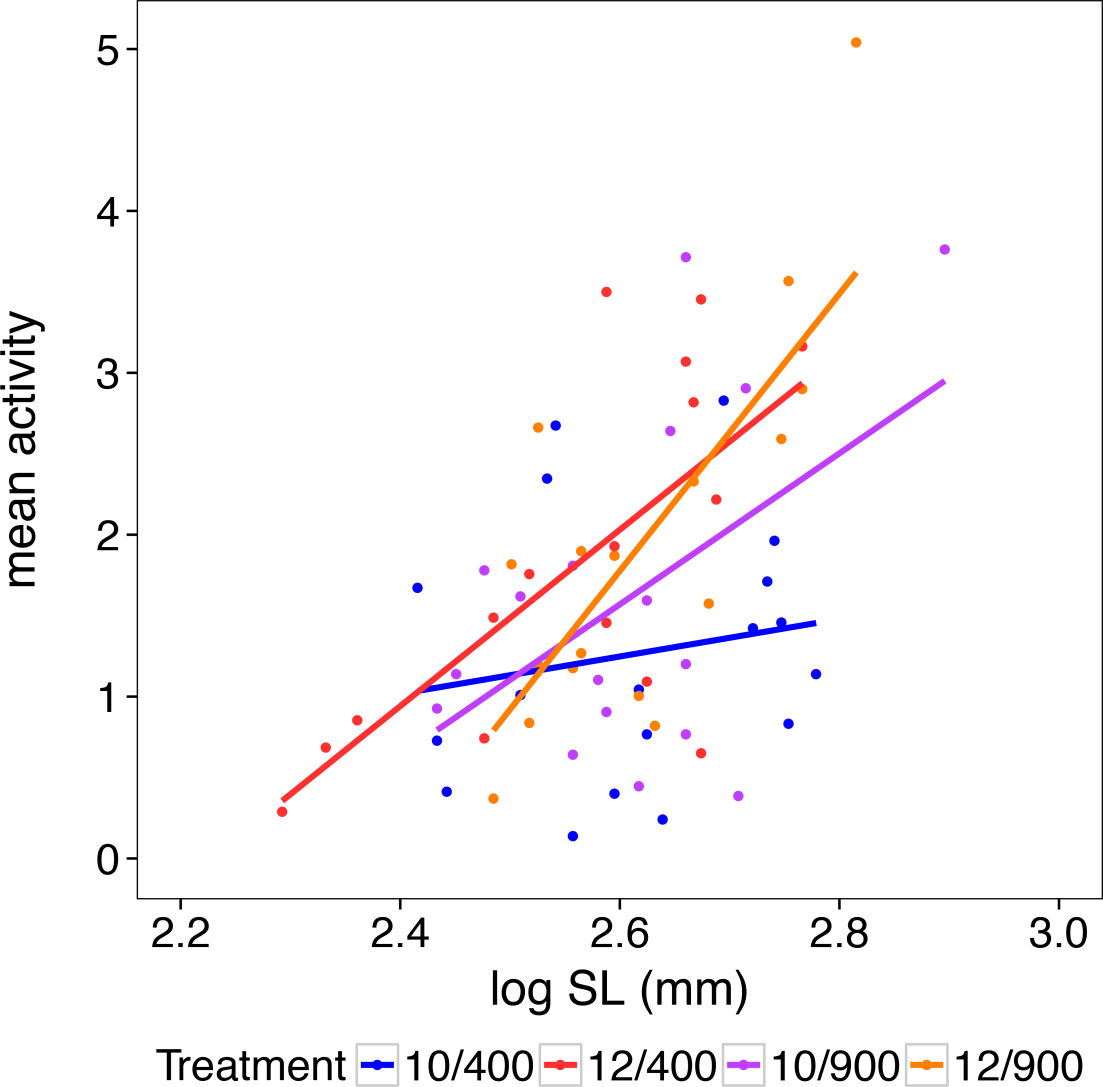
Swimming activity plotted over log standard length (SL) (mm) of herring larvae reared in one of the four temperature/CO_2_ treatment combinations (10/400, 10/900, 12/400 and 12/900) for 28–30 days prior to the measurements. The lines show the linear correlation between swimming activity and standard length for each treatment.

The activity of the larvae at the end of the experiment (28–30 DPH) was higher in 12/400 and 12/900, and also increased significantly with larval length (Fig 4; Table 2; S3 Table). The significant interaction of these two factors can be seen as an increasing temperature effect on the activity of longer larvae. No significant effect of CO_2_ on activity was detected.

### Developmental stages

**Fig 5:**
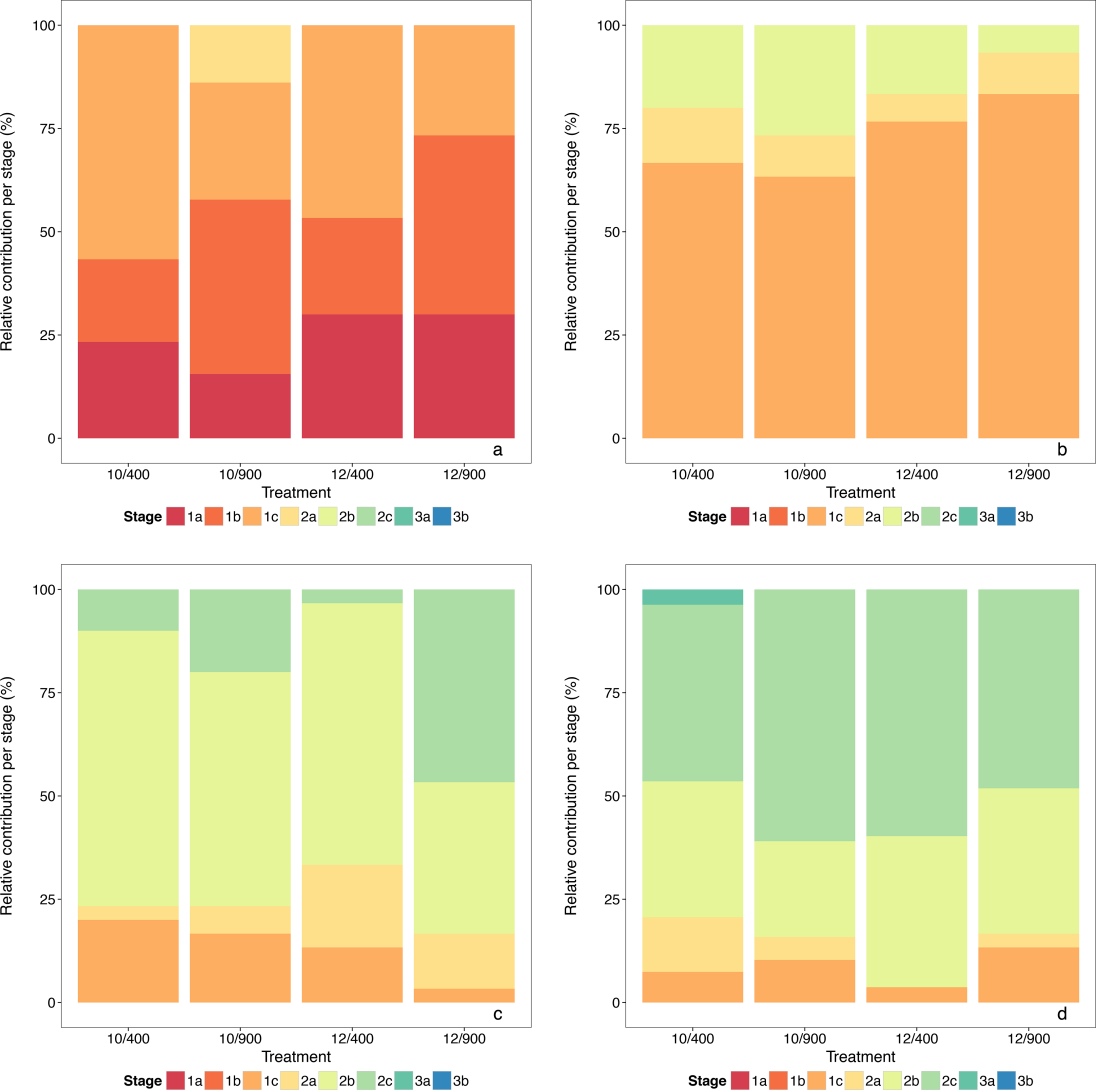
**Relative contribution per developmental stage and treatment for selected sampling days**: (a) 2 DPH, (b) 12 DPH, (c) 22 DPH and (d) 32 DPH.

The development of the larvae was categorized by determining the Doyle stages. A similar development was found in all treatments with the majority of the larvae reaching stage 2c at the end of the experiment (Fig 5). In phase I a significant interaction of temperature and time was detected, being reflected in slightly more developed larvae at 2 and 12 DPH in the 10°C treatments (Table 2).

## Discussion

### Effect of temperature

Although a direct negative effect of warming on Atlantic herring is thought to be unlikely due to the broad thermal tolerance [60], and both temperatures in the experiment were in the preferred range of herring [37,61], a negative effect of higher temperature on survival and instantaneous growth rate was found in this study. The temperature effects detected in the first phase of the experiment on instantaneous growth rate and survival may originate from different response times in yolk utilization [62] and the switch from endogenous to exogenous feeding mode [60]. After this critical first-feeding stage, the general agreement in the literature states that increasing growth is related to higher prey densities and temperatures within the thermal window of the species [60]. One possible reason for the negative temperature effects observed in this study could be increased energy demand at elevated temperatures correlated to increased metabolic rates [63,64], but may also be due to increased activity in the warmer temperature causing higher metabolic rates. As elevated swimming activity in warm water was detected in this study, such a mechanism could be plausible.

The positive effect of temperature on growth, regardless of the prey densities, described in an individual based model for herring [65], is different to our findings, but may be explained by lower prey densities in our study than specified in the model. Usually studies on the effect of temperature on larval growth are performed at *ad libitum* food densities, so the energy required for processes such as growth and metabolism is provided [9] and a positive effect of temperature on growth can be observed [66–68]. The low food levels in the current study limited energy supply, and an increased energy demand in the higher temperature may not have been met. When comparing growth of larvae in the current study to those in Fox et al. [69], similar larval sizes were found in their low food treatment at the same age and temperature, indicating that energy availability was limiting growth in the current study. The effect of low food availability is also supported by the low values of G_i_, resembling bad feeding conditions [70–72] and the delayed larval development in the warm treatment. Therefore, the negative effect of higher temperature is likely a combined direct effect of temperature, as well as increased energy demand. The relatively high G_i_ values found in our study right after hatch most likely relate to the endogenous feeding of the larvae on yolk [60], while the slight increase in growth potential to the end of our study cannot be explained by absolute higher food densities, but may result from increasing encounter rates [73] and improvement in hydrodynamic constraints for feeding [74].

### Effect of CO_2_

Herring larval dry weight was negatively impacted at CO_2_ levels projected for the end of the century (~900 μatm CO_2_), though this effect was only significant in the 10°C treatment and during the first phase of our study. These findings are in concordance with the literature [30,32], and may result from an increased energy demand for osmoregulation during the first days after hatch [75]. For example, Frommel et al. [32] found a negative effect on growth in dry weight and suspected altered organ functionality in the pancreas, liver and kidneys, as underlying mechanisms, while a positive effect on growth was assumed to indicate altered pathways in protein and lipid biosynthesis [18]. Both of these mechanisms may affect larval growth efficiency and survival. The interaction of low energy supply needs to be taken into account, which calls for a more thorough investigation into how effects of different temperatures and elevated CO_2_ concentrations interact at different food levels. The fact that larval herring swimming activity (this study) and swimming kinematics [34] were unaffected by CO_2_ fits with recent findings that many temperate species appear behaviourally robust to increased CO_2_ concentrations [34,58,76–79], despite the negative effects on behaviour that have been reported in several tropical fishes (e.g. [80]). It is unclear why temperate species would be more robust to higher CO_2_ levels than tropical species. It could be due to temperate fish living in more variable environments (e.g. seasonal changes in temperature, oxygen and *p*CO_2_, food density, nutrients), which may require higher ability for phenotypic plasticity than species in less variable environments. This apparent difference between tropical and temperate species may also be an artefact due to differences between studies (e.g. methodological differences, researcher and publication biases, “the decline effect” where early publications on a topic typically overestimate the effect sizes, or phylogenetic differences of the species used from different geographical areas.

Distinct negative effects of OA on survival were found for the important forage fish Atlantic silverside [81] and two populations of the economically important Atlantic cod [20], while in our study survival of Atlantic herring larvae showed contrasting effects depending on the temperature, indicating interspecific differences. A negative CO_2_ effect on survival was detected at 12°C, but a positive effect was detected at 10°C, which calls for a careful interpretation of the data and further research on this topic. Differences in survival between CO_2_ treatments ranged between 1–2 days, which is close to the detection limit of survival rates in our study, since dead larvae were sampled on a daily basis. In general, the effect of CO_2_ was small compared to the effect of temperature in our study, as can be seen in the small effect sizes of the CO_2_ treatments.

Ocean warming has been suggested to increase total energy expenditure for metabolism [82], while ocean acidification has been hypothesized to mainly increase energy expenditure for osmoregulation [75]. Limited energy uptake due to low prey availability, combined with an increased energy demand, may result in less energy available in total for growth and maintenance. Reduced growth rates at higher temperatures in herring have also been reported by Høie et al. [50]. In general, survival can be matched to the growth potential in this study, with larvae in poor condition at a higher risk for starvation [72]. Additionally, the risk of starvation may increase at elevated temperatures, thereby lowering survival, as shown for two age groups of herring larvae [83]. Probably owing to the increased risk of starvation, one of the replicates in the warmer treatment of our study experienced a high mortality at 21 DPH and afterwards showed a strong increase in growth. Two possible explanations for this scenario are an increase in food items per individual or selection for larvae with a lower starvation potential. A selection for phenotypes with better growth at unfavourable prey availability has been shown in the field for larval Atlantic cod *G*. *morhua* [84] and in mesocosms for larval herring [70]. An increased energy demand, at elevated temperature or CO_2_ conditions, may thus have the potential to select for specific phenotypes and could especially interact with altered energy supply in terms of food availability.

### Conclusion

Although there was an indication for a negative CO_2_ effect on larval dry weight in our study, the overall result is that herring larvae were tolerant to end of the century CO_2_ conditions (Hypothesis 1). This was especially true when the larvae's response was compared to the negative impact of elevated temperatures due to low food supply (Hypothesis 2), with impacts on instantaneous growth rate and survival. A synergistic effect of elevated temperatures and CO_2_ concentrations (Hypothesis 3) was indicated for herring larval dry weight and survival, but calls for more detailed investigation, with a focus on selection processes. The comparatively small effect of end of the century CO_2_ concentrations on herring larval performance might be explained by the abiotic conditions already experienced through their life cycle as a bottom spawning species. The diverse results on possible effects of OA on the performance of this economically important species reported in the literature could be explained by differences in biological responses depending on applied OA scenarios, population and life stages. These results also support the propositions that Atlantic herring may be tolerant to future OA scenarios. For a full understanding of the potential impacts of OA on fish larvae, indirect effects via food web interactions will need to be considered and may have the potential to additionally affect performance of early life stages in fish.

## Supporting information

S1 Table Number of larvae per sampling. Number of larvae per replicate (Tank) and sampling day in days post hatch (DPH) used for the respective analysis (SL = standard length, DW = dry weight, Gi = growth potential, Doyle = developmental stage; Swimming activity was only measured once at the end).
(DOCX)

S2 Table Complete results for repeated-measures ANOVA. Outcome of the best fitting repeated-measures ANOVA model for the different parameters with the respective degrees of freedom (DF), F-values and p-values. The factors listed are additive for the described models, e.g. Length~Time+Temp+CO_2_+Temp*CO_2_.
(DOCX)

S3 Table Complete results for ANCOVA. Outcome of the best fitting ANCOVA model for swimming activity with the respective likeliness of fit (R^2^), degrees of freedom (DF), F-values and p-values. The factors listed are additive for the described model.
(DOCX)

S1 Fig Swimming activity over time. Mean (±sd) swimming activity per minute for four treatment combinations of temperature (10°C vs. 12°C) and CO_2_ (400 μatm vs. 900 μatm *p*CO_2_), measured over a 20 minute time interval.
(PDF)
