# Supplementary material for: Growth performance and survival of larval Atlantic herring, under the combined effects of elevated temperatures and CO2
Source: PLoS One. 2018 Jan 25;13(1):e0191947. doi: 10.1371/journal.pone.0191947 (PMC5785030; doi:10.1371/journal.pone.0191947)
Supplement: S1 Table — Number of larvae per replicate (Tank) and sampling day in days post hatch (DPH) used for the respective analysis (SL = standard length, DW = dry weight, Gi = growth potential, Doyle = developmental stage; Swimming activity was only measured once at the end). (DOCX) [file pone.0191947.s001.docx]

S1 Table: Number of larvae per replicate (Tank) and sampling day in days post hatch (DPH) used for the respective analysis (SL=standard length, DW=dry weight, Gi=growth potential, Doyle=developmental stage; Activity was only measured once at the end).

| DPH | Replicate | Treatment | n SL | n DW | n Gi | n Stage | n Activity |
| --- | --- | --- | --- | --- | --- | --- | --- |
| 2 | 1 | 12/400 | 10 | 10 | 10 | 12 |  |
|  | 2 | 10/900 | 10 | 10 | 10 | 10 |  |
|  | 3 | 10/400 | 10 | 10 | 10 | 10 |  |
|  | 4 | 12/900 | 10 | 10 | 10 | 10 |  |
|  | 5 | 12/400 | 10 | 10 | 10 | 10 |  |
|  | 6 | 10/400 | 10 | 10 | 10 | 10 |  |
|  | 7 | 12/900 | 10 | 10 | 10 | 10 |  |
|  | 8 | 10/900 | 10 | 10 | 10 | 10 |  |
|  | 9 | 12/400 | 10 | 10 | 10 | 10 |  |
|  | 10 | 10/900 | 10 | 10 | 10 | 10 |  |
|  | 11 | 10/400 | 10 | 10 | 10 | 10 |  |
|  | 12 | 12/900 | 10 | 10 | 10 | 10 |  |
| 7 | 1 | 12/400 | 10 | 10 | 10 | 10 |  |
|  | 2 | 10/900 | 10 | 10 | 10 | 10 |  |
|  | 3 | 10/400 | 10 | 10 | 10 | 10 |  |
|  | 4 | 12/900 | 10 | 10 | 10 | 10 |  |
|  | 5 | 12/400 | 10 | 10 | 10 | 10 |  |
|  | 6 | 10/400 | 10 | 10 | 10 | 10 |  |
|  | 7 | 12/900 | 10 | 10 | 10 | 10 |  |
|  | 8 | 10/900 | 10 | 10 | 10 | 10 |  |
|  | 9 | 12/400 | 10 | 10 | 10 | 10 |  |
|  | 10 | 10/900 | 10 | 10 | 10 | 10 |  |
|  | 11 | 10/400 | 10 | 10 | 10 | 10 |  |
|  | 12 | 12/900 | 10 | 10 | 10 | 10 |  |
| 12 | 1 | 12/400 | 10 | 10 | 10 | 10 |  |
|  | 2 | 10/900 | 10 | 10 | 10 | 10 |  |
|  | 3 | 10/400 | 10 | 10 | 10 | 10 |  |
|  | 4 | 12/900 | 10 | 10 | 10 | 10 |  |
|  | 5 | 12/400 | 10 | 10 | 10 | 10 |  |
|  | 6 | 10/400 | 10 | 10 | 10 | 10 |  |
|  | 7 | 12/900 | 10 | 10 | 10 | 10 |  |
|  | 8 | 10/900 | 10 | 10 | 10 | 10 |  |
|  | 9 | 12/400 | 10 | 10 | 10 | 10 |  |
|  | 10 | 10/900 | 10 | 10 | 10 | 10 |  |
|  | 11 | 10/400 | 10 | 10 | 10 | 10 |  |
|  | 12 | 12/900 | 10 | 10 | 10 | 10 |  |
| 17 | 1 | 12/400 | 10 | 10 | 10 | 10 |  |
|  | 2 | 10/900 | 10 | 10 | 10 | 10 |  |
|  | 3 | 10/400 | 10 | 10 | 10 | 10 |  |
|  | 4 | 12/900 | 10 | 10 | 10 | 10 |  |
|  | 5 | 12/400 | 10 | 10 | 10 | 10 |  |
|  | 6 | 10/400 | 10 | 10 | 10 | 10 |  |
|  | 7 | 12/900 | 10 | 10 | 10 | 10 |  |
|  | 8 | 10/900 | 10 | 10 | 10 | 10 |  |
|  | 9 | 12/400 | 10 | 10 | 10 | 10 |  |
|  | 10 | 10/900 | 10 | 10 | 10 | 10 |  |
|  | 11 | 10/400 | 10 | 10 | 10 | 10 |  |
|  | 12 | 12/900 | 10 | 10 | 10 | 10 |  |
| 22 | 1 | 12/400 | 10 | 10 | 10 | 10 |  |
|  | 2 | 10/900 | 10 | 10 | 10 | 10 |  |
|  | 3 | 10/400 | 10 | 10 | 10 | 10 |  |
|  | 4 | 12/900 | 10 | 10 | 10 | 10 |  |
|  | 5 | 12/400 | 10 | 10 | 10 | 10 |  |
|  | 6 | 10/400 | 10 | 10 | 10 | 10 |  |
|  | 7 | 12/900 | 10 | 10 | 10 | 10 |  |
|  | 8 | 10/900 | 10 | 10 | 10 | 10 |  |
|  | 9 | 12/400 | 10 | 10 | 10 | 10 |  |
|  | 10 | 10/900 | 10 | 10 | 10 | 10 |  |
|  | 11 | 10/400 | 10 | 10 | 10 | 10 |  |
|  | 12 | 12/900 | 10 | 10 | 10 | 10 |  |
| 27 | 1 | 12/400 | 10 | 10 | 10 | 10 |  |
|  | 2 | 10/900 | 10 | 10 | 10 | 10 |  |
|  | 3 | 10/400 | 10 | 10 | 10 | 10 |  |
|  | 4 | 12/900 | 10 | 10 | 10 | 10 |  |
|  | 5 | 12/400 | 9 | 10 | 10 | 5 |  |
|  | 6 | 10/400 | 10 | 10 | 10 | 9 |  |
|  | 7 | 12/900 | 10 | 10 | 10 | 10 |  |
|  | 8 | 10/900 | 10 | 10 | 10 | 8 |  |
|  | 9 | 12/400 | 10 | 10 | 10 | 10 |  |
|  | 10 | 10/900 | 10 | 10 | 10 | 7 |  |
|  | 11 | 10/400 | 10 | 10 | 10 | 9 |  |
|  | 12 | 12/900 | 10 | 10 | 10 | 10 |  |
| 28 | 1 | 12/400 |  |  |  |  | 6 |
|  | 2 | 10/900 |  |  |  |  | 6 |
|  | 3 | 10/400 |  |  |  |  | 6 |
|  | 4 | 12/900 |  |  |  |  | 6 |
|  | 5 | 12/400 |  |  |  |  |  |
|  | 6 | 10/400 |  |  |  |  |  |
|  | 7 | 12/900 |  |  |  |  |  |
|  | 8 | 10/900 |  |  |  |  |  |
|  | 9 | 12/400 |  |  |  |  |  |
|  | 10 | 10/900 |  |  |  |  |  |
|  | 11 | 10/400 |  |  |  |  |  |
|  | 12 | 12/900 |  |  |  |  |  |
| 29 | 1 | 12/400 |  |  |  |  |  |
|  | 2 | 10/900 |  |  |  |  |  |
|  | 3 | 10/400 |  |  |  |  |  |
|  | 4 | 12/900 |  |  |  |  |  |
|  | 5 | 12/400 |  |  |  |  | 6 |
|  | 6 | 10/400 |  |  |  |  |  |
|  | 7 | 12/900 |  |  |  |  |  |
|  | 8 | 10/900 |  |  |  |  |  |
|  | 9 | 12/400 |  |  |  |  | 6 |
|  | 10 | 10/900 |  |  |  |  |  |
|  | 11 | 10/400 |  |  |  |  |  |
|  | 12 | 12/900 |  |  |  |  |  |
| 30 | 1 | 12/400 |  |  |  |  |  |
|  | 2 | 10/900 |  |  |  |  |  |
|  | 3 | 10/400 |  |  |  |  |  |
|  | 4 | 12/900 |  |  |  |  |  |
|  | 5 | 12/400 |  |  |  |  |  |
|  | 6 | 10/400 |  |  |  |  | 6 |
|  | 7 | 12/900 |  |  |  |  | 6 |
|  | 8 | 10/900 |  |  |  |  | 6 |
|  | 9 | 12/400 |  |  |  |  |  |
|  | 10 | 10/900 |  |  |  |  | 6 |
|  | 11 | 10/400 |  |  |  |  | 6 |
|  | 12 | 12/900 |  |  |  |  | 6 |
| 32 | 1 | 12/400 | 10 | 10 | 10 | 7 | 6 |
|  | 2 | 10/900 | 10 | 10 | 10 | 8 | 6 |
|  | 3 | 10/400 | 10 | 10 | 10 | 7 | 6 |
|  | 4 | 12/900 | 10 | 10 | 10 | 9 | 6 |
|  | 5 | 12/400 | 10 | 10 | 10 | 6 | 5 |
|  | 6 | 10/400 | 10 | 10 | 10 | 9 | 6 |
|  | 7 | 12/900 | 10 | 10 | 10 | 10 | 6 |
|  | 8 | 10/900 | 10 | 10 | 10 | 8 | 6 |
|  | 9 | 12/400 | 10 | 10 | 10 | 10 | 5 |
|  | 10 | 10/900 | 10 | 10 | 10 | 9 | 6 |
|  | 11 | 10/400 | 10 | 10 | 10 | 5 | 6 |
|  | 12 | 12/900 | 10 | 10 | 10 | 10 | 6 |
| DPH | Tank |  | n SL | n DW | n sRD | n Doyle | n Activity |
