# Supplementary material for: Growth performance and survival of larval Atlantic herring, under the combined effects of elevated temperatures and CO2
Source: PLoS One. 2018 Jan 25;13(1):e0191947. doi: 10.1371/journal.pone.0191947 (PMC5785030; doi:10.1371/journal.pone.0191947)
Supplement: S2 Table — Outcome of the best fitting repeated-measures ANOVA model for the different parameters with the respective degrees of freedom (DF), F-values and p-values. The factors listed are additive for the described models, e.g. Length~Time+Temp+CO2+Temp*CO2. (DOCX) [file pone.0191947.s002.docx]

S2 Table: Outcome of the best fitting repeated-measures ANOVA model for the different parameters with the respective degrees of freedom (DF), F-values and p-values. The factors listed are additive for the described models, e.g. Length~Time+Temp+CO_2_+Temp*CO_2_.

| Parameter | Period | Temperature | Factor | DF | F-value | p-value |
| --- | --- | --- | --- | --- | --- | --- |
| Length | Whole | 10°C / 12°C | Temp | 8, 71 | 0.130 | 0.73 |
|  |  |  | CO_2_ |  | 0.550 | 0.48 |
|  |  |  | **Time** |  | **244.780** | **<0.0001** |
|  |  |  | Temp*CO_2_ |  | 2.560 | 0.15 |
|  | Phase 1 | 10°C / 12°C | Temp | 8, 47 | 0.050 | 0.83 |
|  |  |  | CO_2_ |  | 0.950 | 0.36 |
|  |  |  | **Time** |  | **192.610** | **<0.0001** |
|  |  |  | Temp*CO_2_ |  | 4.380 | 0.07 |
|  | Phase 2 | 10°C / 12°C | **Time** | 23 | **6.610** | **<0.05** |
| Weight | Whole | 10°C / 12°C | Temp | 8, 71 | 0.110 | 0.75 |
|  |  |  | CO_2_ |  | 0.850 | 0.38 |
|  |  |  | **Time** |  | **199.630** | **<0.0001** |
|  |  |  | Temp*CO_2_ |  | 4.450 | 0.07 |
|  | Phase 1 | 10°C / 12°C | Temp | 8, 47 | 0.002 | 0.96 |
|  |  |  | CO_2_ |  | 2.167 | 0.18 |
|  |  |  | **Time** |  | **89.200** | **<0.0001** |
|  |  |  | **Temp*CO_2_** |  | **10.986** | **<0.05** |
|  | Phase 2 | 10°C / 12°C | **Time** | 23 | **13.660** | **<0.05** |
| Instantaneous growth rate | Whole | 10°C / 12°C | **Time** | 71 | **89.245** | **<0.0001** |
|  | Phase 1 | 10°C / 12°C | Temp | 8, 47 | 0.047 | 0.83 |
|  |  |  | CO_2_ |  | 3.091 | 0.11 |
|  |  |  | **Time** |  | **609.724** | **<0.0001** |
|  |  |  | **Temp*Time** |  | **7.413** | **<0.01** |
|  | Phase 2 | 10°C / 12°C | **Time** | 10, 22 | **60.783** | **<0.001** |
|  |  |  | Temp |  | 2.769 | 0.13 |
|  |  |  | Temp*Time |  | 4.220 | 0.05 |
| Development | Whole | 10°C / 12°C | **Time** | 71 | **678.71** | **<0.0001** |
|  | Phase 1 | 10°C / 12°C | **Time** | 8,44 | **617.78** | **<0.0001** |
|  |  |  | Temp |  | 1.01 | 0.34 |
|  |  |  | CO_2_ |  | 0.870 | 0.38 |
|  |  |  | **Temp*Time** |  | **6.560** | **<0.05** |
|  |  |  | Time* CO_2_ |  | 1.890 | 0.18 |
|  |  |  | Temp* CO_2_ |  | 0.870 | 0.38 |
|  |  |  | Temp*Time* CO_2_ |  | 3.280 | 0.08 |
|  | Phase 2 | 10°C / 12°C | **Time** | 8, 20 | **14.50** | **<0.01** |
|  |  |  | Temp |  | 0.48 | 0.51 |
|  |  |  | CO_2_ |  | 0.020 | 0.88 |
|  |  |  | Temp*Time |  | 2.970 | 0.10 |
|  |  |  | Time*CO2 |  | 0.180 | 0.68 |
|  |  |  | Temp*CO2 |  | 0.020 | 0.89 |
|  |  |  | Temp*Time*CO2 |  | 2.400 | 0.14 |
