# Supplementary material for: Growth performance and survival of larval Atlantic herring, under the combined effects of elevated temperatures and CO2
Source: PLoS One. 2018 Jan 25;13(1):e0191947. doi: 10.1371/journal.pone.0191947 (PMC5785030; doi:10.1371/journal.pone.0191947)
Supplement: S3 Table — Outcome of the best fitting ANCOVA model for swimming activity with the respective likeliness of fit (R2), degrees of freedom (DF), F-values and p-values. The factors listed are additive for the described model. (DOCX) [file pone.0191947.s003.docx]

S3 Table: Outcome of the best fitting ANCOVA model for swimming activity with the respective likeliness of fit (R^2^), degrees of freedom (DF), F-values and p-values. The factors listed are additive for the described model.

| Parameter | Period | Temperature | R^2^ | Factor | DF | F-value | p-value |
| --- | --- | --- | --- | --- | --- | --- | --- |
| Swimming  activity | Final | 10°C / 12°C | **0.36** | **SL** | 6, 56 | **25.90** | **<0.001** |
|  |  |  |  | **Temp** |  | **7.12** | **<0.01** |
|  |  |  |  | CO_2_ |  | 0.36 | 0.55 |
|  |  |  |  | Temp* CO_2_ |  | 1.67 | 0.20 |
|  |  |  |  | SL* CO_2_ |  | 2.70 | 0.11 |
|  |  |  |  | **SL*Temp** |  | **5.42** | **<0.05** |
