# Supplementary figures and images for: Growth performance and survival of larval Atlantic herring, under the combined effects of elevated temperatures and CO2
Source: PLoS One. 2018 Jan 25;13(1):e0191947. doi: 10.1371/journal.pone.0191947 (PMC5785030; doi:10.1371/journal.pone.0191947)

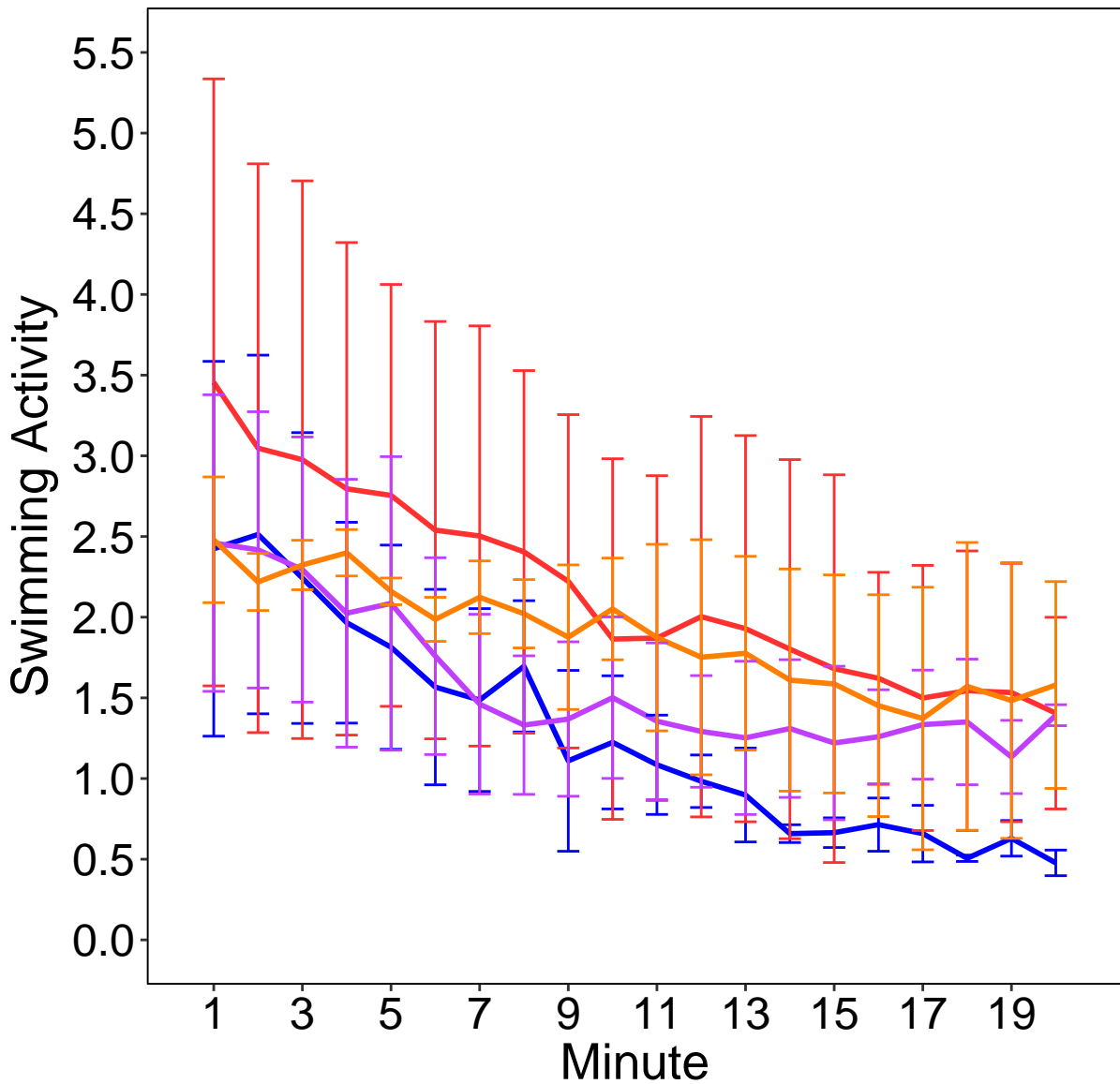

Supplement: S1 Fig — Mean (±sd) swimming activity per minute for four treatment combinations of temperature (10°C vs. 12°C) and CO2 (400 μatm vs. 900 μatm pCO2), measured over a 20 minute time interval. (PDF) [file pone.0191947.s004.pdf]
